# Tinea Incognito Imitating Pustular Psoriasis in a Psoriatic Patient Following Immunosuppressive Therapy: Case Report and Narrative Review

**DOI:** 10.3390/jcm15072743

**Published:** 2026-04-04

**Authors:** Maksymilian Markwitz, Nina Łabędź, Natalia Welc, Krzysztof Kanabaj, Monika Bowszyc-Dmochowska, Honorata Kubisiak-Rzepczyk, Aleksandra Dańczak-Pazdrowska

**Affiliations:** 1 Department of Dermatology, Poznan University of Medical Sciences, 60-355 Poznan, Poland; 2 Doctoral School, Poznan University of Medical Sciences, 60-812 Poznan, Poland; 3 Cutaneous Histopathology and Immunopathology Section, Department of Dermatology, Poznan University of Medical Sciences, 60-355 Poznan, Poland

**Keywords:** tinea incognito, psoriasis, immunosuppressive therapy

## Abstract

Tinea incognito is an atypical form of dermatophytosis caused by previous use of topical or systemic immunosuppressive therapy, most often corticosteroids. Modification of the clinical presentation frequently leads to diagnostic delay and misdiagnosis, especially in patients with concomitant chronic inflammatory skin diseases such as psoriasis. We present a narrative review of the literature on tinea incognito in patients with psoriasis during immunosuppressive therapy. We screened 386 abstracts and included 16 comparable case reports focusing on tinea incognito occurring in patients with psoriasis or during antipsoriatic treatment. The review summarizes clinical presentations, diagnostic challenges, and therapeutic approaches reported in the literature. Additionally, we present a clinical case of a 27-year-old man with a long history of plaque psoriasis treated with methotrexate and cyclosporine. The patient developed rapidly progressive skin lesions with pustular features and further deterioration despite systemic antipsoriatic therapy. Initial mycological examinations were negative. Histopathological examination revealed a chronic purulent perifollicular inflammatory process with extension into the subcutaneous tissue. The correct diagnosis was confirmed after a repeat skin biopsy with periodic acid–Schiff and Grocott staining and fungal culture of the skin tissue, which revealed *Trichophyton rubrum*. The review highlights that clinical features are often nonspecific and may overlap with inflammatory dermatoses. This underscores the need for a high index of clinical suspicion for fungal infection in atypical or refractory psoriatic lesions. It also emphasizes the importance of repeated mycological and histopathological examinations to achieve an accurate diagnosis, avoid inappropriate escalation of immunosuppression, and enable timely antifungal treatment.

## 1. Introduction

Dermatophyte infections are among the most common superficial fungal diseases worldwide, affecting approximately 20–25% of the global population [1]. Psoriasis affects approximately 2–3% of individuals and frequently requires long-term topical or systemic immunosuppressive therapy [1]. The coexistence of these conditions is therefore relatively common and may pose significant diagnostic challenges.

Dermatophytosis of glabrous skin typically presents as annular, erythematous lesions with peripheral scaling and central clearing [2]. However, these features may be altered or obscured by topical or systemic immunosuppressive therapy. The term tinea incognito refers to a dermatophyte infection with an atypical clinical presentation resulting from prior use of corticosteroids or other immunomodulatory agents that suppress inflammation while allowing fungal proliferation [3]. As a consequence, classic dermatophyte features may be diminished or absent, frequently leading to delayed or incorrect diagnosis.

*Tinea incognito* is particularly relevant in patients with long-standing psoriasis receiving immunosuppressive therapy. Impaired cell-mediated immunity, especially involving Th1 and Th17 pathways, may increase susceptibility to dermatophyte infection and alter its clinical presentation [4,5]. In this context, dermatophytosis may mimic or coexist with psoriatic lesions, contributing to diagnostic uncertainty. In patients with chronic psoriasis, new or atypical eruptions may be attributed to disease exacerbation or treatment failure rather than to coexisting infection. Consequently, fungal infections may be overlooked, leading to inappropriate escalation of immunosuppressive therapy and further disease progression [6].

Despite increasing awareness, *tinea incognito* remains underrecognized and underreported. Current evidence is largely limited to case reports and small case series, suggesting that atypical dermatophyte infections may contribute to diagnostic delay and suboptimal management in immunosuppressed patients [3,7]. Early mycological examination is therefore essential, particularly in atypical or treatment-resistant cases.

In this article, we present a narrative review of the literature on tinea incognito in patients with psoriasis receiving immunosuppressive therapy, focusing on its clinical spectrum, pathophysiology, diagnostic pitfalls, and management. We also report a clinical case of tinea incognito in a patient with plaque psoriasis treated with methotrexate and cyclosporine. Our aim is to highlight the importance of incorporating mycological assessment, including repeated testing when necessary, into the diagnostic algorithm for atypical or treatment-resistant psoriatic lesions to improve patient outcomes.

## 2. Methodology

**Figure 1:**
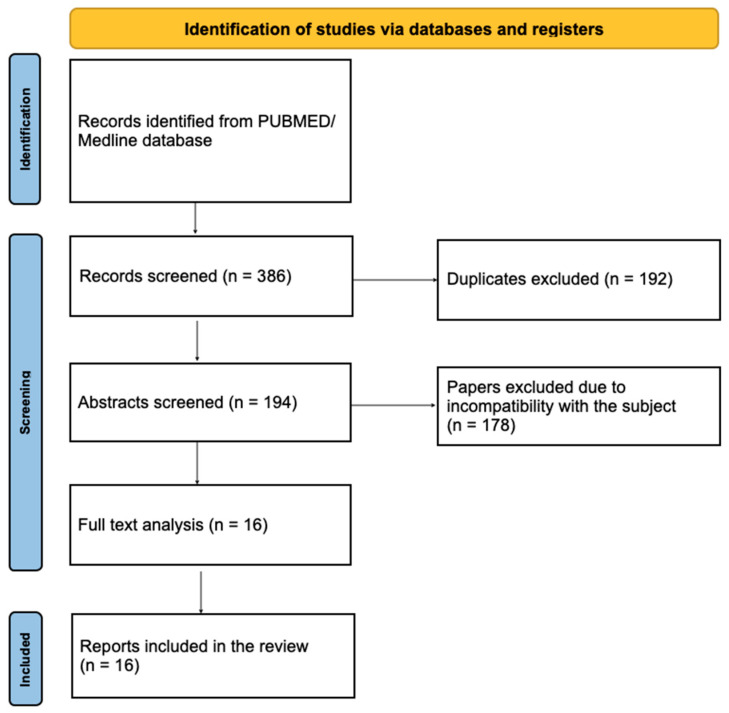
The screening and paper selection process, according to the PRISMA guideline.

We performed a literature review in the PubMed database to identify similar cases reported over the past ten years, using the search terms “psoriasis” and “dermatophytosis,” “psoriasis” and “tinea,” and “psoriasis” and “tinea incognito.” Only articles connected directly to the subject of tinea incognito during psoriasis treatment or clinically mimicking pustular psoriasis were included in the review. A total of 386 abstracts were screened, and 16 case reports with comparable diagnostic and therapeutic characteristics were selected for analysis and summarized in Table 1. The full screening process following PRISMA guidelines is presented in Figure 1.

## 3. Results

Findings from the literature review, including descriptions of the diagnosis, treatment, and clinical picture, are presented in Table 1.

## 4. Clinical Case

A 27-year-old male patient was admitted to the Department of Dermatology due to a sudden and progressive worsening of skin lesions in the course of psoriasis. The deterioration had been observed for approximately three months prior to admission. According to the medical history, the patient had a 12-year history of psoriasis. Initially, psoriasis treatment consisted exclusively of topical glucocorticosteroids. The patient’s family history for psoriasis was negative. The patient denied any chronic comorbidities and reported no regular medications. Until hospitalization, the patient had remained under the care of a regional outpatient dermatology clinic, where good disease control had been maintained throughout most of the disease course. However, approximately six months before hospital admission, a local exacerbation of psoriatic lesions was noted. As a result, the treatment strategy was modified at the outpatient clinic, and systemic immunotherapy with methotrexate was initiated at a dose of 12.5 mg subcutaneously once weekly. This therapy was continued for 10 weeks without clinical improvement, and the skin lesions further deteriorated. Due to the lack of response and progressive worsening, a decision was made to introduce another systemic treatment. Consequently, cyclosporine was initiated at a dose of 2.80 mg/kg body weight, three weeks prior to hospitalization. During therapy, a rapid deterioration of the dermatological condition was observed.

**Figure 2:**
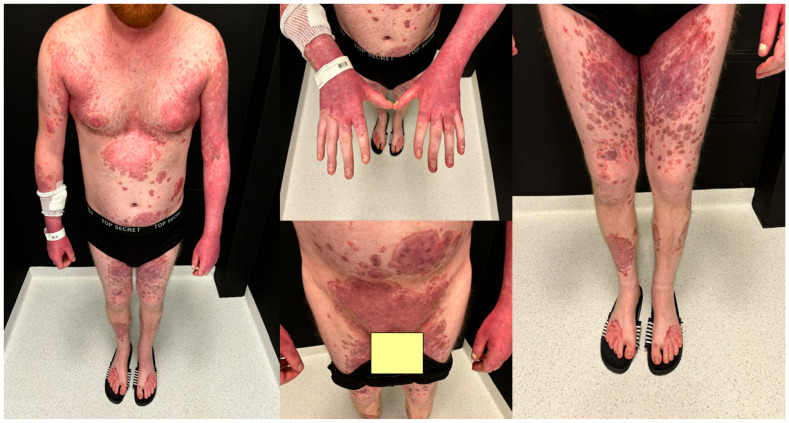
Dermatological examination at admission.

**Figure 3:**
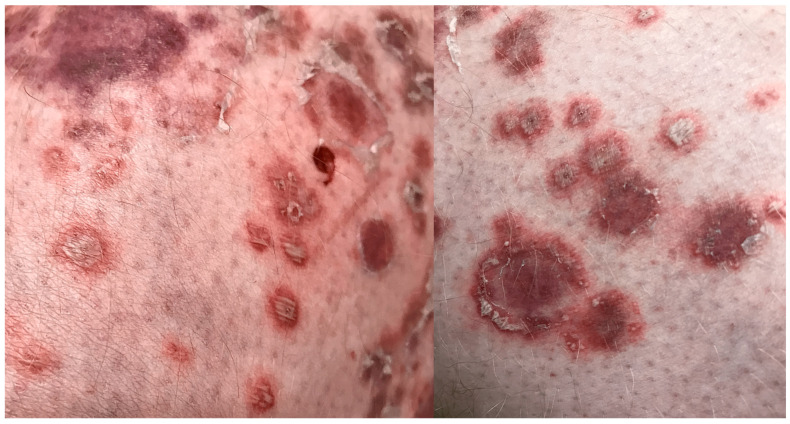
Close-up clinical images showing sparse pustular lesions on the upper and lower limbs. Some pustules appear partially dried or covered with crusts.

A dermatologist from a regional outpatient clinic referred the patient to the dermatology department with an initial clinical suspicion of pustular psoriasis due to the lack of response to immunosuppressive therapy and rapid clinical deterioration. On dermatological examination at admission, extensive confluent erythematous areas with polycyclic and annular morphology were present over almost the entire body surface. Single papular and pustular lesions were also observed. The pustules were superficial, sparse, and most clearly visible on the upper and lower limbs. At admission, in most areas, they appeared partially dried or covered with crusts. In the context of the patient’s history of psoriasis, the presence of these pustular lesions on an erythematous background initially raised suspicion of pustular psoriasis rather than typical dermatophytosis. The most severe lesions were noted in the genital area, the intergluteal cleft, the interdigital spaces of the feet, and the nail units. Written informed consent was obtained from the patient for the use of clinical photographic documentation presented in Figure 2. The pustular lesions are shown in the high-resolution close-up images in Figure 3.

Laboratory blood tests after admission to the hospital revealed leukocytosis (16 × 10^3^/µL) with neutrophilia (11 × 10^3^/µL). Inflammatory markers were significantly elevated, with C-reactive protein (CRP) at 134 mg/L and procalcitonin at 1.5 ng/mL. Mild elevations of liver enzymes were noted (ALT 56 IU/L, AST 63 IU/L). Repeated direct mycological examinations and fungal cultures were performed five times, all of which were negative. Examination under a Wood’s lamp was conducted five times and was also negative.

**Figure 4:**
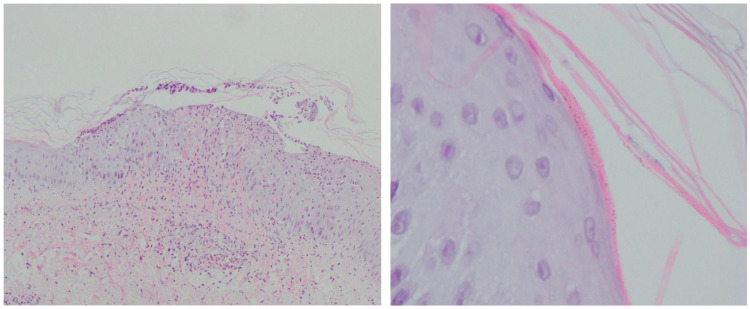
Histopathological examination. On the left, edematous epidermis infiltrated by neutrophils and erythrocytes forming a subcorneal neutrophilic pustule (H + E stain, original magnification ×10). On the right, fungal spores and hyphae within the stratum corneum (H + E stain, original magnification ×60); blue arrows indicate fungal spores and hyphae.

Two skin biopsies were obtained for histopathological evaluation and were independently reviewed by a board-certified dermatopathologist. The first biopsy demonstrated a chronic purulent perifollicular inflammatory process extending into the subcutaneous tissue. Due to clinical suspicion of immunosuppression-induced cutaneous mycosis, the material was submitted for additional fungal staining (PAS and Grocott). The second specimen revealed a skin fragment with a purulent perifollicular inflammatory infiltrate in the mid-dermal layers. No pustules or microscopic features of psoriasis were identified in the epidermis. Also, no fungal elements were visible on routine hematoxylin and eosin staining of the thickened stratum corneum or hair follicles; however, given the clinical suspicion, further PAS and Grocott staining was requested. Unfortunately, the staining results were negative and did not reveal fungal elements in the examined material. The patient was discharged home with a presumptive diagnosis of pustular psoriasis and was prescribed acitretin at a dose of 10 mg orally, along with topical therapy. Due to the absence of clinical improvement, the patient was readmitted to the dermatology ward for further diagnostic evaluation. A third skin biopsy was obtained. Histopathological examination revealed a mildly thickened epidermis with central neutrophilic infiltration forming a neutrophilic pustule in the upper epidermal layers. Neutrophilic exocytosis was accompanied by erythrocyte infiltration of the epidermis. Within the stratum corneum, elongated, isolated structures suggestive of fungal hyphae were observed and required confirmation with PAS or Grocott staining. The histological picture was considered consistent with tinea corporis and required differentiation from pustular psoriasis with a hemorrhagic component if fungal infection was not confirmed (Figure 4).

**Figure 5:**
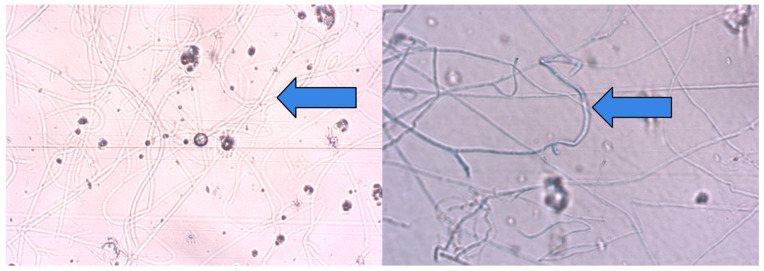
Fungal filaments identified on direct microscopic examination of the fourth skin biopsy. Blue arrows indicate fungal filaments.

Additional staining with PAS and Grocott revealed sparse fungal spores within the stratum corneum, as well as fungal hyphae. Also, it was decided to perform direct microscopic examination of the fourth skin biopsy due to positive PAS and Grocott staining, which confirmed the presence of fungal filaments, as shown in Figure 5.

**Figure 6:**
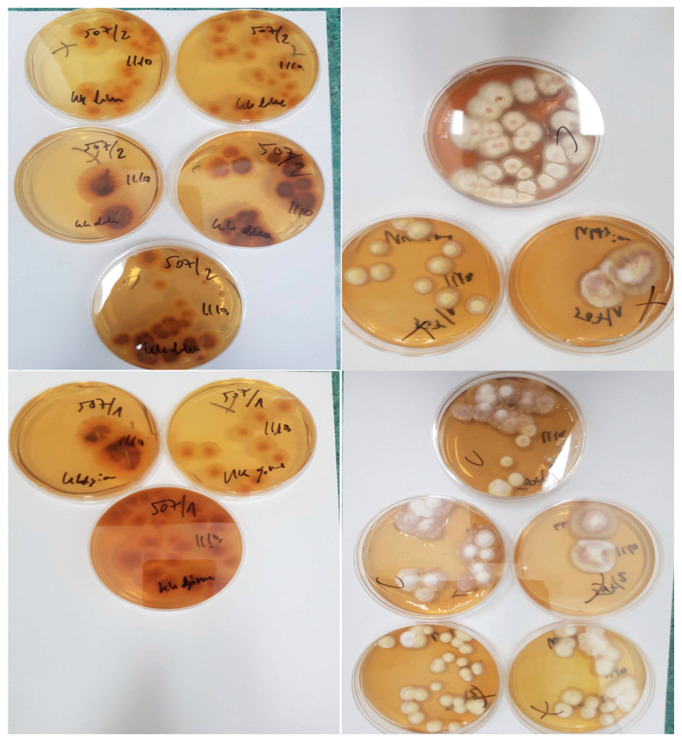
Fungal growth was observed on Sabouraud dextrose agar after 21 days of incubation. The colonies were well-developed, circular, and showed a white-to-cream-colored, fluffy surface with a slightly raised center. The reverse side of the colonies exhibited characteristic yellow-to-yellow-brown pigmentation. Based on the macroscopic morphology and subsequent microscopic evaluation, the isolate was identified as *Trichophyton rubrum* with yellow pigment.

After 21 days of incubation, the fungal culture on Sabouraud dextrose agar demonstrated visible colony growth. The isolate exhibited characteristic macroscopic features on both the obverse and reverse sides of the culture medium, including a white-to-cream-colored, fluffy surface and a yellowish pigmentation on the reverse. Based on these macroscopic morphological characteristics, the organism was identified as *Trichophyton rubrum*, which produces a yellow pigment, as seen in Figure 6; see Appendix A for the method description.

**Figure 7:**
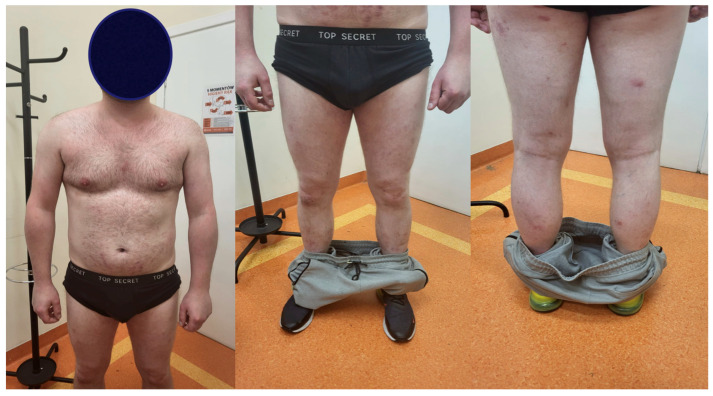
Clinical appearance of the skin lesions after 1 month of antifungal therapy, showing improvement of the dermatological condition.

**Figure 8:**
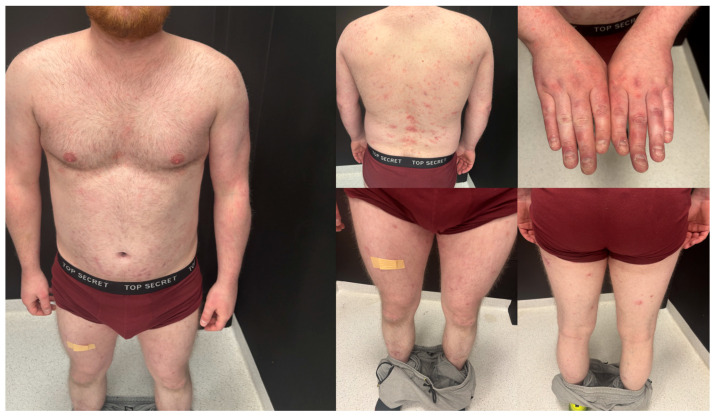
Clinical appearance of the skin lesions after 3 months of antifungal therapy, showing radical improvement of the dermatological condition.

Antifungal therapy was administered for three months. The therapy included topical 10% isoconazole applied twice daily and oral terbinafine at 250 mg once daily. The patient was evaluated at follow-up visits after 1 month and 3 months. At the 1-month outpatient follow-up, an improvement in the dermatological condition was observed Figure 7. A clear normalization of inflammatory parameters, including leukocytosis, C-reactive protein (CRP), and procalcitonin, was observed, and therefore, the treatment was continued. At the subsequent follow-up after an additional 2 months, a significant improvement in the dermatological condition was noted, as seen in Figure 8.

The patient remained under continuous follow-up at our dermatology clinic. Twelve months after completion of antifungal therapy, a new exacerbation of the dermatological condition was observed. However, the clinical presentation was typical of plaque psoriasis and no longer suggestive of fungal infection.

## 5. Discussion

Superficial fungal infections of glabrous skin are a very common dermatological problem worldwide, while tinea incognito may account for approximately 40% of all dermatophytoses [24,25]. Despite its frequent occurrence and global distribution, the true incidence of this condition is likely underestimated, as many cases are misdiagnosed or remain unreported. The lack of a clearly established diagnostic standard for suspected tinea incognito contributes to the condition’s frequent underrecognition in dermatological practice [25]. Consequently, delayed or incorrect diagnosis often leads to inappropriate treatment, most commonly anti-inflammatory therapy, which further modifies the clinical presentation and promotes progression of tinea incognito [26]. The precise frequency of tinea incognita is unknown, whereas the reported prevalence of dermatophytosis is 40% [27,28]. Most cases are probably underreported or misdiagnosed. Dermatophytosis more often develops in high-humidity, high-temperature areas and may occur in children and adults, with males more commonly affected [29]. Sometimes, the relatively low severity of the symptoms may also contribute to the current lack of proper epidemiological studies [29]. The mild disease severity and rapid clinical resolution following topical therapy often lead to symptom remission, and such patients are neither further investigated nor reported. Therefore, the cases described in the literature (Table 1) predominantly represent severe grades of disease courses requiring hospitalization, which allowed for comprehensive diagnostic evaluation and accurate diagnosis.

Tinea incognito is an atypical manifestation of dermatophytosis caused by changes in the host’s inflammatory response due to immunosuppressive therapies, most commonly topical or systemic corticosteroids, but also topical calcineurin inhibitors and systemic immunomodulatory agents. The term tinea incognito was first used in 1968 to describe dermatophyte infections with altered clinical morphology, in which classical features of tinea are partially or completely masked, often leading to misdiagnosis and inappropriate escalation of anti-inflammatory treatment [30]. Across published series and case reports, *Trichophyton rubrum* remains the most frequently identified causative organism of tinea incognito, reflecting its global predominance in dermatophytoses and its anthropophilic nature [31,32]. Other commonly implicated species include *Trichophyton mentagrophytes*, Epidermophyton floccosum, and zoophilic dermatophytes such as *Microsporum canis* [31,32,33]. However, some studies have identified *Microsporum canis* as the leading causative pathogen [34]. In recent years, increasing attention has been drawn to *Trichophyton indotineae*, an emerging, often terbinafine-resistant species associated with extensive, treatment-refractory, and highly inflammatory disease, including corticosteroid-modified presentations [32]. Tinea incognito affects people of all ages, but it is less common in infants and individuals over 75 years of age [34]. The most commonly affected sites include the trunk, followed by the face, eyelids, scalp, and intertriginous areas [31,35,36]. Involvement of distant sites, such as the feet and nails, is not uncommon and may result from autoinoculation, particularly in patients receiving prolonged immunosuppressive therapy [36]. The clinical presentation of tinea incognito varies widely and is influenced by the type, potency, and duration of immunosuppressive therapy. In contrast to classical dermatophytosis, lesions typically lack a sharply demarcated, scaly border and central clearing, and instead present as ill-defined erythematous plaques or patches, often with minimal surface scaling. Lesions are frequently extensive, asymmetric, and rapidly progressive [37]. A notable feature of tinea incognito is the presence of papules and pustules within the lesions. Pustules may be primary, related to follicular dermatophyte invasion, or secondary, resulting from bacterial superinfection, a pattern increasingly reported in recent case studies [37,38]. Pruritus may be reduced or temporarily suppressed, but itchy lesions are still commonly reported [37]. Due to its variable morphology, tinea incognito often mimics other inflammatory dermatoses, such as eczema, psoriasis, rosacea, cutaneous lupus erythematosus, seborrheic dermatitis, or bacterial folliculitis, leading to diagnostic errors and delays in appropriate mycological evaluation.

Patients with pre-existing psoriasis represent a particular diagnostic challenge, as dermatophyte infection may either mimic psoriatic exacerbation or coexist with psoriasis [11,13,21]. Several reports have described the simultaneous occurrence of psoriasis and dermatophytosis, and fungal infection itself may occasionally trigger psoriatic lesions through the Koebner phenomenon, in which cutaneous inflammation or trauma induces the development of psoriatic plaques in predisposed individuals [21,37]. Consequently, distinguishing between pustular psoriasis and dermatophytosis may be difficult, particularly in patients receiving immunosuppressive therapy [13,37]. In the present case, the diagnostic process was guided by the atypical clinical course. The patient showed progressive worsening despite systemic antipsoriatic therapy; annular and polycyclic lesions were present in intertriginous and acral areas. Initial biopsies did not show classical histopathological features of psoriasis. The patient was initially discharged with a presumptive diagnosis of pustular psoriasis, which further reflects the diagnostic difficulty in such cases. The final diagnosis was established only after repeated sampling and histopathological examination with PAS and Grocott staining, which demonstrated fungal elements subsequently confirmed by fungal culture.

Immunosuppressive agents that may promote the development of tinea incognito also constitute a standard therapeutic approach in patients with moderate-to-severe psoriasis [39]. In the presented patient, immunosuppressive treatment was administered in accordance with current dermatological recommendations [37]. According to the available literature, the most frequently described factors leading to the development of tinea incognito are topical corticosteroids (TCS), whose uncontrolled or prolonged use may mask the typical clinical presentation of dermatophytosis, promote its spread, and delay correct diagnosis [39,40]. The literature also highlights topical calcineurin inhibitors, such as tacrolimus and pimecrolimus, which, by modulating the host immune response, may lead to an atypical course of dermatophyte infection [41]. Topical corticosteroids, widely available in many countries as over-the-counter medications, are often the first-line treatment for nonspecific inflammatory skin lesions and are also initiated by physicians without access to specialized dermatological diagnostic tools, potentially delaying recognition of fungal infection [42,43]. Additionally, the use of combination formulations containing topical corticosteroids together with antifungal and/or antibacterial agents, as well as over-the-counter preparations, may paradoxically increase the risk of infection recurrence rather than provide a preventive effect [39,40,44]. Systemic glucocorticosteroids are also reported to trigger tinea incognito; however, this occurs much less frequently than with topical corticosteroids [43]. In recent years, reports have also emerged describing tinea incognito in patients treated with biologic agents, including TNF-alpha inhibitors and IL-17 inhibitors [45]. In these cases, the main pathogenic mechanism appears to be increased susceptibility to dermatophytosis and modification of the clinical course of infection during immunomodulation, rather than the classical mechanism of inflammatory masking observed with topical corticosteroid use [46]. Reports of tinea incognito during cyclosporine therapy are very limited, and the available data remain scarce. However, this does not necessarily mean that the condition is rare. Tinea incognito is often underdiagnosed and underreported, and its frequency in clinical practice is likely higher than suggested by the literature. Therefore, the value of our case is not based on its uniqueness, but on showing an important diagnostic problem in psoriasis patients receiving immunosuppressive therapy.

In the analyzed cases (*n* = 16), most patients were initially diagnosed with plaque psoriasis (*n* = 13), generalized pustular psoriasis (*n* = 2), and subcorneal pustular dermatosis/IgA pemphigus (*n* = 1). From a morphological point of view, all skin lesions were plaques. Pustules were found in nine patients, scaling in 11, and four had an annular pattern. The most common locations of the lesions were: trunk (*n* = 10), buttocks (*n* = 7), arms, face and neck (*n* = 5), shoulders (*n* = 4), legs, and scalp (*n* = 3). Only one individual had ears, feet, and an upper lip affected. Two of the patients had nail lesions. In our case report, plaques and pustules were observed, most prominently on the upper and lower limbs. Alopecia was present in only one of them. The most frequent drug that incited the tinea was topical corticosteroids with or without calcipotriol (*n* = 11). Some patients exhibited a flare after receiving biological treatment (*n* = 4), mostly with TNF-alpha inhibitors or IL-17 inhibitors (adalimumab, infliximab, ixekizumab, and bimekizumab; one case each).

The use of appropriate diagnostic tools is crucial for the recognition of dermatophyte infections. A lack of corticosteroid efficacy should be a warning sign that diagnostic tests should be initiated. Usually, the diagnosis of tinea incognito requires confirmation by mycological examination performed after prior discontinuation of topical glucocorticosteroids [3,44]. To date, no scientific studies have clearly defined the number of days that should elapse between discontinuation of topical glucocorticosteroids and the performance of a mycological examination. In most publications, this interval is described only as “a few days,” without specifying the exact number of days required to obtain a reliable mycological result [44]. Based on our clinical experience, mycological examination is most commonly repeated approximately 5 days after cessation of topical glucocorticosteroid therapy. In contrast, following prior use of antifungal agents, including both topical preparations and combination products containing an antifungal drug with a glucocorticosteroid, it should be noted that the 4-week interval required to obtain a reliable mycological examination is at least 2 weeks and, in some cases, even 4 weeks. This is related to prolonged suppression of dermatophyte growth and an increased risk of false-negative results [47,48].

On the other hand, numerous scientific studies have demonstrated that histopathological examination is more sensitive than fungal culture and direct microscopic examination with potassium hydroxide (KOH) [49]. In recent years, dermatophytoses of the glabrous skin and face have been reported to frequently exhibit unusual, nonspecific, or inconspicuous histopathological features [49]. The histopathological appearance of tinea corporis and tinea faciei is highly heterogeneous and, in most cases, dominated by nonspecific changes, including parakeratosis, a loose basket-weave pattern of the stratum corneum, spongiosis, and papillary dermal edema, which may mimic other inflammatory dermatoses [49,50]. Classical features considered to be more specific for dermatophytoses, including the presence of neutrophils in the stratum corneum, compact orthokeratosis, and the so-called “sandwich” sign, are observed relatively infrequently and cannot be regarded as sole diagnostic criteria [50,51]. It should be emphasized that fungal hyphae are detectable on routine haematoxylin and eosin-stained sections in only a minority of cases, even in the presence of a typical clinical presentation [49]. Therefore, in the histopathological evaluation of suspected inflammatory lesions of the glabrous skin and face, the routine use of special stains such as periodic acid–Schiff and Grocott methenamine silver, together with mycological culture of skin scrapings, is recommended, as these approaches significantly increase the detection of fungal elements and reduce the risk of false-negative results and diagnostic delay [42,47,49].

In the present case, the diagnostic process evolved over several stages and was influenced by ongoing immunosuppressive therapy. Direct mycological examination of skin scrapings was performed five times during systemic treatment with methotrexate and subsequently cyclosporine; however, all results were negative. Fungal cultures obtained during this period also did not identify the causative pathogen. Two initial skin biopsies did not reveal fungal elements on routine histopathological examination, and PAS and Grocott staining were also negative. Because of persistent clinical suspicion and progressive worsening of the skin lesions, further diagnostic sampling was performed after readmission. The third biopsy revealed structures suggestive of fungal hyphae within the stratum corneum on routine histopathological examination, and subsequent PAS and Grocott staining demonstrated sparse fungal spores and hyphae. Direct microscopic examination performed on material obtained from the fourth skin biopsy confirmed the presence of fungal filaments. The diagnosis was subsequently confirmed by fungal culture, identifying *Trichophyton rubrum*. Currently, molecular methods such as polymerase chain reaction (PCR) can also be used to diagnose fungal infections [51,52]. These techniques are rapid and highly sensitive. However, their main limitations are high cost and limited availability outside academic settings [53].

Extensive inflammatory skin involvement may be associated with systemic laboratory abnormalities, including leukocytosis and elevated inflammatory markers such as C-reactive protein (CRP) and procalcitonin [54,55]. Although such findings are more commonly associated with bacterial infections or systemic inflammatory conditions, severe and widespread cutaneous inflammation alone may also cause significant laboratory deviations. In the available literature on tinea incognito and dermatophytosis, elevations of leukocyte count, CRP, or procalcitonin are rarely discussed and remain poorly characterized [56]. In the presented case, markedly elevated inflammatory parameters were observed in the absence of a confirmed bacterial infection, suggesting that extensive fungal infection with an intense inflammatory response may contribute to systemic laboratory abnormalities. Importantly, following the initiation of appropriate antifungal therapy, a clear normalization of leukocyte count, CRP, and procalcitonin levels was observed, supporting a causal relationship between the underlying cutaneous infection and the systemic inflammatory response [57].

## 6. Conclusions

The increasing reliance on a “treat to diagnose” approach, together with the widespread use of multi-component topical preparations containing corticosteroids, antibiotics, and antifungals, contributes to the misdiagnosis of dermatophytosis by masking infections and promoting atypical clinical presentations. Maintaining a high index of suspicion for fungal infection is therefore essential in atypical, treatment-resistant, or rapidly progressive inflammatory dermatoses, particularly in patients receiving immunosuppressive therapy. To reduce diagnostic delays and patient morbidity, definitive diagnosis should be prioritized using direct microscopic examination (KOH), fungal culture, and, when necessary, histopathological evaluation with special stains. Early recognition of dermatophytosis may prevent inappropriate escalation of immunosuppressive therapy. In addition, increasing awareness among healthcare professionals, especially general practitioners who often initiate first-line treatment, is crucial to limit iatrogenic modification of fungal infections and to ensure timely and accurate management.

## 7. Future Directions

In daily clinical practice, particular attention should be paid to identifying the causes of atypical and unexpected skin lesions, especially in patients receiving immunosuppressive therapy or in those treated chronically with topical corticosteroids. Prospective studies are needed to evaluate optimal diagnostic algorithms, including the appropriate timing of mycological examinations after discontinuation of corticosteroids or antifungal agents to reduce the risk of false-negative results and diagnostic delays. Further research should also assess the role of advanced diagnostic methods, such as molecular techniques, in the early detection of dermatophyte infections presenting with atypical or pustular clinical manifestations. In addition, larger clinical studies are required to better characterize the epidemiology, clinical spectrum, and risk factors of tinea incognito in immunosuppressed patients. Improved understanding of these aspects may contribute to the development of standardized diagnostic pathways and help prevent unnecessary escalation of immunosuppressive therapy. These findings highlight an underrecognized and likely underreported diagnostic problem that may lead to inappropriate treatment decisions.

## Figures and Tables

**Table 1:** Literature review of tinea incognito cases developed during psoriasis treatment or treated.

Authors	Sex, Age of the Patient	Treatment of Unrecognized Tinea Incognito	Clinical Picture	Diagnosis	Treatment
Eichhoff [8]	Male, 46	-	Multiple partially annular lesions with subtle scaling and multiple pustules on the arms, legs, buttocks, and groin six weeks after the beginning of the treatment with adalimumab.	Direct examination and skin scraping culture: *Trichophyton rubrum*.	Fluconazole.
Starace [9]	Male, 17	Topical GCS with calcipotriol.	The dorsum of the foot presented a wide erythematous area, with well-demarcated scaling margins and some follicular papules.	Direct examination and skin scraping culture: *Trichophyton rubrum*.	Terbinafine and topical antifungal.
Kalkan [10]	Female, 89	Topical GCS.	Plaques, accompanied by pustules and desquamation on the back and front of the trunk for approximately one year.	Direct examination: fungal hyphae.	Systemic and topical antifungal.
Serarslan [11]	Female, 11	Topical GCS, oral antihistamine.	Erythematous, sharply demarcated lesions with pustules on the upper trunk, shoulders, and right side of the face.	Direct examination, culture: *Trichophyton rubrum*.	Terbinafine and topical antifungal.
Chu [12]	Male, 60	Topical GCS.	Irregular infiltrative erythema on the face, right ear, scalp, neck, shoulder, trunk, and left arm, and small pustules scattered on the scalp and upper lip.	Culture, PCR: *Trichophyton interdigitale*.	Oral itraconazole and topical antifungal.
Emelianov [13]	Male, 44	Topical GCS and calcipotriol.	Widely spread itchy erythrosquamous papules, plaques, and onychodystrophy affecting all of the patient’s fingers and toenails during treatment with ixekizumab.	Histopathology, skin scrapings culture: *Trichophyton rubrum*.	Terbinafine and topical antifungal.
Norimatsu [14]	Male, 75	Topical GCS with calcipotriol.	Erythematopustular lesions on the lumbar region and thighs.	Grocott, PAS staining, tissue culture: *Trichophyton rubrum*.	Oral fosravuconazole and topical antifungal.
Kawakami [15]	Female, 3	Topical GCS.	Extensive erythema, with multiple pustules on the chest, widespread rash on the face and chin.	Direct examination, PCR genotyping: *Trichophyton mentagrophytes* var. *interdigitale*.	Terbinafine and topical antifungal.
Segal [16]	Male, 57	Topical GCS with calcipotriol, phototherapy.	Annular erythematous scaly lesions with central sparing coalescing into diffuse plaques with areas of scale and eschar on the arms, torso, head, and neck.	PAS staining, culture: *Trichophyton rubrum*.	Oral fluconazole.
Khosravi [17]	Male, 30	Topical GSC and oral retinoid.	Large, severe erythematous plaques covered by papulovesicular and pustular lesions in the pubis, cruris, and buttock regions.	Direct examination and skin scraping culture: *Trichophyton mentagrophytes* var. *mentagrophytes*.	Oral itraconazole and topical antifungal.
Đorđević Betetto [18]	Male, 68	Topical GCS and calcipotriol.	Large erythematous confluent plaques with well-demarcated scaly borders with annular configuration on the trunk, buttocks, and lower extremities.	Histopathology: solitary hyphae, PAS staining (+). Direct examination: negative.	Systemic and topical terbinafine.
Rallis [19]	Male, 58	Topical pimecrolimus.	Extensive, erythematous, dry, scaly plaques with a peripheral, scaly, raised border with lichenification, excoriations, and follicular pustules on the groins, lower abdomen, and medial aspects of the thighs.	Direct examination and skin scraping culture: *Trichophyton rubrum*.	Oral itraconazole and topical antifungal.
Cosio [20]	Male, 36	-	An annular, erythematous scaly plaque with a serpiginous border on the neck, right shoulder, and frontotemporal left area developed during treatment with bimekizumab.	Direct examination, PCR: *Trichophyton tonsurans*.	Terbinafine and topical antifungal.
Asnani [21]	Male, 45	Topical GCS and increased dosage of oral methotrexate.	Cutaneous examination revealed widespread erythematous plaques with grayish-white scales over the neck, abdomen, groin folds, buttocks, and back after increased dosage of methotrexate.	Direct examination, histopathology: *Trichophyton mentagrophytes*.	Oral itraconazole and topical antifungal.
Balaguer-Franch [22]	Female, 79	-	Erythematous scaly plaques, with well-defined edges and superficial excoriations, on the scalp, shoulder blades, and retroauricular region, loss of hair follicles in the frontotemporal hairline and the eyebrows, violaceous–erythematous papules on the thighs, subungual hyperkeratosis with chromonychia during infliximab treatment.	Culture: *Trichophyton* spp.	Terbinafine.
Diruggiero [23]	Male, 64	Topical GCS.	Excoriated plaques with surrounding scale on buttocks, hips, lower back, abdomen, upper chest, and neck, lichenifications with linear excoriations on the bilateral lower extremities and forearms.	Direct examination: fungal hyphae.	Terbinafine and topical antifungal.

## Data Availability

The data presented in this study are available on request from the corresponding author.

## Appendix A

Direct mycological assessment was performed using samples obtained from the collected diagnostic material. Prior to examination, specimens were processed with a potassium hydroxide solution (10–20%) combined with dimethyl sulfoxide (40%). The prepared samples were then analyzed for the presence of fungal structures using light and phase contrast microscopy at magnifications of ×200 and ×400. Image acquisition and documentation were carried out with ZEN Microscopy Software version 3.9. To further enhance visualization of fungal elements, selected specimens were stained with calcofluor and examined under fluorescence microscopy, allowing detection of fungal hyphae based on their characteristic fluorescent properties. For definitive species identification, the material was inoculated onto Sabouraud dextrose agar supplemented with chloramphenicol and actidione and incubated at both 25 °C and 37 °C for up to six weeks. Dermatophyte identification was subsequently achieved through evaluation of colony morphology and microscopic features, including the structure of hyphae and spores.
